# The Hidden Cost of COVID-19: Focus on Antimicrobial Resistance in Bloodstream Infections

**DOI:** 10.3390/microorganisms11051299

**Published:** 2023-05-16

**Authors:** Giulia Micheli, Flavio Sangiorgi, Francesca Catania, Marta Chiuchiarelli, Federico Frondizi, Eleonora Taddei, Rita Murri

**Affiliations:** 1 Dipartimento di Sicurezza e Bioetica, Università Cattolica del Sacro Cuore, 00168 Rome, Italy; 2 Dipartimento di Scienze di Laboratorio e Infettivologiche, Fondazione Policlinico Universitario Agostino Gemelli IRCCS, 00168 Rome, Italy

**Keywords:** COVID-19, antimicrobial resistance, AMR, bloodstream infection, BSI, MRSA, VRE, 3GCR-E, CRE, DTR-PA, CR-Ab

## Abstract

Antibiotic resistance is one of the greatest growing public health threats and a worldwide priority. According to the WHO, drug-resistant diseases may cause 10 million deaths a year by 2050 and have a substantial impact on the global economy, driving up to 24 million people into poverty. The ongoing COVID-19 pandemic has exposed the fallacies and vulnerability of healthcare systems worldwide, displacing resources from existing programs and reducing funding for antimicrobial resistance (AMR) fighting efforts. Moreover, as already seen for other respiratory viruses, such as flu, COVID-19 is often associated with superinfections, prolonged hospital stays, and increased ICU admissions, further aggravating healthcare disruption. These events are accompanied by widespread antibiotic use, misuse, and inappropriate compliance with standard procedures with a potential long-term impact on AMR. Still, COVID-19-related measures such as increasing personal and environmental hygiene, social distancing, and decreasing hospital admissions could theoretically help the AMR cause. However, several reports have shown increased antimicrobial resistance during the COVID-19 pandemic. This narrative review focuses on this “twindemic”, assessing the current knowledge of antimicrobial resistance in the COVID-19 era with a focus on bloodstream infections and provides insights into the lessons learned in the COVID-19 field that could be applied to antimicrobial stewardship initiatives.

## 1. Introduction

Almost a century after the first antibiotic molecule discovery, antibiotics are becoming less effective due to antimicrobial resistance (AMR) which represents one of the most dangerous threats to human existence. According to recent research on the global burden of AMR in 2019, up to 5 million deaths were associated with bacterial AMR with almost 1.30 million deaths directly attributable to this phenomenon [1]. Not only AMR has effects on mortality, but multidrug-resistant infection survivors have lasting consequences as it caused 192,000 disability-adjusted life years (DALYs) in 2019 [1].

Past estimates predicted the impact of AMR on human life up to 10 million deaths a year and a cumulative 100 trillion USD loss of economic output by 2050 [2].

AMR was responsible for 670,000 drug-resistant bacterial infections in the European area in 2020 and 2.8 million antibiotic-resistant infections in the United States of America (USA) in 2017, according to the most recent assessments by the European Centre for Disease Prevention and Control (ECDC) and Centers for Disease Control and Prevention (CDC) for the United States of America (USA) [3,4].

Therefore, AMR spread is an urgent topic that needs global coordinated action and intervention. For this reason, surveillance programs are vital to understanding the impact, informing risk assessments, and mitigating AMR effects through alertness and preparedness. Several programs are in place worldwide and the Global Antimicrobial Resistance Surveillance System (GLASS), promoted by World Health Organization (WHO), stands out as the first system that has made it possible to report official national data on antimicrobial resistance in targeted microbes and antimicrobial consumption in a standardized and consistent manner on a global scale [5]. On the European scale, pathogens currently included in the ECDC surveillance are *Escherichia coli*, *Klebsiella pneumoniae*, *Pseudomonas aeruginosa*, *Acinetobacter* spp., *Staphylococcus aureus*, *Enterococcus faecalis* and *Enterococcus faecium*, which are also part of the WHO critical and high priority pathogens for research and development of new antibiotics [4,6].

In this scenario, the ongoing COVID-19 pandemic has exposed the fallacies and the vulnerability of healthcare systems worldwide. According to the special report “COVID-19 US impact on antimicrobial resistance”, for example, a reduction in detection and reporting of AMR data was documented during the pandemic, along with an alarming increase in resistant infections during hospitalization [7]. The prevalence of these germs was deeply affected by the pandemic, as the report documented a rise of carbapenem-resistant *A. baumannii* up to 78%, carbapenem-resistant Enterobacterales up to 35%, vancomycin-resistant *Enterococcus* spp. 14%, multidrug-resistant *P. aeruginosa* 32%, and methicillin-resistant *S. aureus* 13%, when compared to pre-pandemic data [7].

This was attributed to changes in healthcare, with deep consequences, short and long-term on the individual and community levels. The CDC report points out possible reasons: patient demographics change with sicker patients at the beginning of the pandemic, reduced community access to healthcare increasing underdiagnosis, misdiagnosis, and over-the-counter prescriptions, overuse of antibiotics, lab supply challenges, testing and treatment.

Some meta-analyses studied antibiotic misuse during the pandemic: for example, Langford et al. found that the overall prevalence of antibiotic use in hospitalized COVID-19 patients was 72.3%, with the highest usage reported in critically ill patients, mostly without a documented bacterial coinfection. Moreover, the study reported a higher mortality risk and longer hospital stays in COVID-19 patients who received antibiotics compared to those who did not [8].

The COVID-19 pandemic has also hindered several infection prevention and control measures, including but not limited to, hand hygiene, cleaning of equipment, patient isolation, and use of personal protective equipment (PPE). As a result, progress made toward combating AMR has suffered a setback. For example, during the first year of the pandemic, there was an increase of at least 15% in both hospital-acquired resistant infections and deaths from 2019 to 2020 [7].

In this context, a recent study from a reference tertiary university hospital in Rome, based on 2534 patients, found that patients admitted to COVID settings had a higher risk of developing bloodstream infections (BSIs) compared to other hospital settings, with the highest risk found in COVID intensive care units (ICU). The prevalence of difficult-to-treat pathogens was also highest in COVID-ICU, with almost half of *S. aureus* isolates showing susceptibility to methicillin, and only a small proportion of *Acinetobacter* spp. isolates being susceptible to carbapenems [9].

Currently, more and more literature reviews are being published describing the link between AMR increase and COVID-19 [8,10,11], but little is known about how this microbiological trend specifically relates to BSI.

This narrative review aims to explore the impact of the COVID-19 pandemic on AMR in bloodstream infections. By evaluating the current literature, we will discuss the potential consequences of antibiotic overuse, and existing strategies to reduce AMR and consider future directions in the management and prevention of such infections.

## 2. Materials and Methods

A literature search was conducted on PubMed and WHO websites to identify articles investigating antimicrobial resistance trends in bloodstream infections with data from the pre-pandemic and/or pandemic periods, that were published from March 2020 to March 2023. We focused the search on bacterial bloodstream infection (BSI) in adults and more precisely articles describing trends about methicillin-resistant *Staphylococcus aureus* (MRSA), vancomycin-resistant *Enterococcus* spp. (VRE), third-generation cephalosporins-resistant (3GCR) and carbapenem-resistant Enterobacterales (CRE), carbapenem-resistant *Acinetobacter baumannii* (CR-Ab), and difficult-to-treat resistant *Pseudomonas aeruginosa* (DTR-PA). The pathogens were determined following the ECDC surveillance program and WHO priority pathogens [4,6], based on their clinical significance in nosocomial settings and their importance as healthcare quality proxies.

For this review, the focused search was performed on the PubMed library database using the following keywords: “COVID-19”, “bloodstream infection” in pair with the specific pathogen (“methicillin-resistant *S. aureus*”, “vancomycin-resistant *Enterococcus* spp.”, “third-generation cephalosporins-resistant Enterobacterales”, “carbapenem-resistant Enterobacterales”, “carbapenem-resistant *A. baumannii*”, and “difficult-to-treat *P. aeruginosa*”). Observational studies, systematic reviews, and metanalysis were included. Studies from both ICU and non-ICU departments were included, along with studies where the setting was unspecified. Both studies that had a comparison group (non-COVID-19 era or non-COVID-19 department) and studies without it were included. The search was limited to results in English language and results from languages other than English were excluded.

## 3. Results

The main findings are resumed in Table 1. In Table 2, possible explanations about AMR changing epidemiology during the COVID-19 pandemic are described, related to classical infection prevention and control measures, and antimicrobial stewardship.

### 3.1. Methicillin-Resistant Staphylococcus aureus (MRSA)

Methicillin-resistant *Staphylococcus aureus* (MRSA) has been for several years a leading cause of infection worldwide, particularly in bacteremia, upper and lower respiratory tract infections, and skin and soft tissue infections. MRSA is considered one of the most important hospital-acquired pathogens, based on its capacity to acquire resistance and a wide number of virulence factors (hemolysins, enterotoxins, and proteases) [12,13].

*S. aureus* is the second most frequent cause of hospital-acquired/health-care-associated infections (HAIs) in the USA, causing up to 12% of all HAIs in 2015–2017 [14].

According to European surveillance data, up to 20% of the *S. aureus* isolates were resistant to at least one of these antibiotics in 2020 (methicillin/MRSA, fluoroquinolones, and rifampicin). However, the European population-weighted mean MRSA percentage (excluding the United Kingdom) was 16.7% in the same period, marking a significantly decreasing trend since 2016 (when the MRSA percentage was 19.3%). Significant differences in MRSA prevalence between countries were observed, with higher rates generally observed in southern and eastern European countries compared to those in northern Europe [4].

Recently, the connection between COVID-19 and bacterial infections has become increasingly investigated and, as already described in past viral pandemics, *S. aureus* has been the principal cause of secondary bacterial infections, significantly increasing mortality rates. For example, in 2020, a retrospective observational case series described 42 cases of *S. aureus* BSI among 2657 COVID-19 patients: the study found that the patients with BSI had a significantly higher 30-day mortality rate compared to those without BSI (54.8% versus 25%) [15].

The incidence of BSI caused by MRSA increased during the pandemic: a retrospective study reported an increase of 1.6 times in MRSA BSI in a multihospital institution in Paris, France [16] while a retrospective and prospective observational epidemiological study in Italy found an increase in all MDR infection rates, including MRSA. The Authors attributed this increase to the fact that, in the pandemic era, access to the emergency department was available to most, if not only, to severely ill patients. This could mean longer periods of hospitalization and a higher HAI rate [17].

A retrospective observational study in Brazil comes to the same result, stating that the increase in MRSA infections in ICU and non-ICU settings was over 46% [18], with peaks in the ordinary ward that reached 100%. A comparative analysis focusing on BSI in COVID-19 patients versus non-COVID-19 patients in ordinary wards found that all staphylococcal BSI were due to MRSA in COVID wards and about only 42% in ordinary wards [19]. In a reference institution in Rome, Italy, as previously reported, there was a significant increase in *S. aureus* BSI incidence during the COVID-19 period compared to a pre-COVID cohort, with MRSA phenotype in more than 43% of these BSI during the pandemic [9].

A systematic review on the impact of COVID-19 on AMR analyzed 22 studies on the matter of incidence of MRSA: the Authors concluded that, even if there are differences based on many factors (epidemiology, study design), the overall trend points towards an increase in the prevalence of MRSA infection [10]. 

On the other hand, early data from retrospective research showed a reduction of MDR BSI during the pandemic period compared to the pre-pandemic era, particularly in MRSA [20], attributing this evidence to the introduction of personal protection devices.

Last, a scoping review in 2021 found 57 patients in 28 articles infected with MRSA and COVID following their complete clinical course (among them 64% were BSI). They found an increase, as well as previously detected by Cusumano et al., in morbidity and mortality [21].

### 3.2. Vancomycin-Resistant Enterococcus spp. (VRE)

*Enterococcus* spp. are one of the most common causes of hospital-associated infections representing the second and third most frequently observed pathogens responsible for HAI in the USA and Europe, respectively. During the last 20 years, vancomycin-resistant Enterococci (VRE) keep increasing in hospitals [22]. Reported mortality rates in enterococcal BSI range from 19% to 48% [23] higher in VRE compared to enterococcal species that are susceptible to vancomycin [24].

There is evidence of an increased incidence of *Enterococcus* spp. BSI in critically ill patients with COVID-19 [25]. Even at the beginning of the pandemic, as early as February 2020, a retrospective observational study documented a significant increase incidence of BSIs in ICU patients, particularly *Enterococcus* spp. BSIs, compared with the previous 2 years [26] (as high as 87/1000 days of ICU stay during early COVID versus during the same period in 2018 (24/1000) or 2019 (19/1000). This finding was confirmed by another study where BSIs were significantly associated with positivity for SARS-CoV-2, mechanical ventilation, and chronic obstructive pulmonary disease [27]. In addition, DeVoe et al. found that COVID-19 patients had a higher incidence of BSI due to *Enterococcus* spp. (unadjusted OR when compared to controls 7.8 (95% CI, 3.6–16.6) for BSI with Enterococcus, but not BSI generally [28].

Higher rates of community and hospital-acquired BSI rates were observed during the pandemic period from March 2020 and September 2021, compared to the pre-pandemic period (July 2019 to February 2020). *Enterococcus* spp. was the second and third most frequent pathogen in community and hospital BSI, respectively [29].

Unfortunately, in these studies, the Authors did not collect data about antibiotic resistance mechanisms.

Few literature reviews and meta-analyses have focused on VRE BSI alone, and evidence regarding VRE infections during the COVID pandemic is often conflicting.

A recent meta-analysis of 23 studies showed that the COVID-19 pandemic was not associated with a change in the incidence density of VRE infections [30]. A recent systematic review included five studies, reporting an increase in VRE infections during the pandemic compared to the pre-pandemic period and 3 studies that, instead, demonstrated a reduction during the pandemic [10].

A retrospective study conducted in Lebanon showed a decrease in isolation density, defined as the number of isolates/1000 patient days (the number of isolates divided by the patient days in a given time multiplied by 1000), of *E. faecium* BSI, and in particular, a significant reduction of 34% of VRE BSI in 2020 (considered as the pandemic period) compared with the previous period from 2015 to 2019 [31].

On the other hand, Jeon et al. showed a significant increase in the prevalence of VRE in clinical samples, which includes not only BSI, during the pandemic period (from March 2020 to September 2021). In contrast, surveillance samples showed a decreased prevalence of VRE (r^2^ = 0.734, *p* = 0.029) [32]. In a prospective cohort study, including almost a hundred healthcare-affiliated hospitals in the USA, it was found an increased relative rate of VRE infection, including BSIs, adjusted for the increased number of COVID-19 hospitalization [33]. In a reference teaching hospital in Rome, Italy, there was a significant increase in E. faecium BSI incidence during the COVID-19 period compared to a pre-COVID cohort and the rate of the VRE phenotype was 38% during the pandemic [9].

Interestingly, Metan et al. conducted a retrospective study comparing the incidence of BSI during the same period in 2 twin hospitals, one served as a pandemic hospital, the other COVID-19-free. They found no differences in infection density rates of VRE BSI, although there was a significant increase in infection density rates of ampicillin-resistant *E. faecium* BSI in the pandemic hospitals compared to COVID-19-free one and compared to a previous period (2018–2019) [34].

A Greek surveillance study confirmed increased isolation of *E. faecium* BSI during a pandemic period, as other studies reported, and showed an increasing non-susceptibility trend for vancomycin and teicoplanin in the period from April 2020 to March 2021, compared to the previous 26 months [35].

An increase in the incidence density of VRE BSI was also noted in a Taiwanese hospital during the first year of the COVID-19 pandemic, compared to the previous two years, despite an increased consumption of 75% alcohol-based disinfectant and persistent efforts for antibiotic stewardship program. However, the Authors also noted a decreasing consumption of chlorhexidine solutions (−4%) and increasing usage of third-generation cephalosporins may favor the occurrence of VRE infections [36].

Finally, a comparative analysis of microbial epidemiology and AMR profiles in BSI among COVID-19 and non-COVID-19 patients in ordinary wards in the same period found that Enterococcal bacteremia was significantly more frequent in COVID-19 patients with a high rate of VRE (19% versus 8.8%). They presumed that prior antibiotic consumption in the community by outpatient physicians or over-the-counter purchases can alter gut flora and facilitate VRE colonization and subsequent infection [19].

### 3.3. Enterobacterales

Enterobacterales are a family of Gram-negative rods, which include *E. coli,* the most prevalent cause of BSI worldwide [37]. Beta-lactamase production is the most prominent mechanism of resistance among Enterobacterales, while third-generation cephalosporins and carbapenems are the main targets of beta-lactamases producing multi-drug resistant (MDR) organisms [38].

Due to its clinical significance, the epidemiology of BSI due to Enterobacterales has been studied since the beginning of the pandemic. A retrospective multicenter study conducted in the UK from 1 April 2017 to 30 April 2020 showed a significant decrease in BSI due to Enterobacterales between the first months of 2020 and the average of the previous years, with no mention of MDR organisms. The Authors speculate that the phenomenon may be due to a combination of “stay at home” orders for patients with fever, early utilization of antibiotics before blood culture withdrawal, and a reduction in abdominal and urinary elective surgery activity [39].

Many reports also dealt with MDR Enterobacterales, mostly including miscellaneous infections rather than BSIs only. Below we describe the reports of BSIs due to third-generation cephalosporins-resistant (3GCR) and carbapenem-resistant Enterobacterales (CRE), due to relevance and rigorous microbiological definition of such infections.

#### 3.3.1. Third-Generation Cephalosporins-Resistant (3GCR) Enterobacterales

A multicenter French study showed a significant increase in the rate of BSIs caused by 3GCR Enterobacterales, particularly ESBL-producing *K. pneumoniae*, in March-April 2020 compared to the same period in 2019. The Authors also noted increased blood culturing and antibiotics utilization [16].

In a tertiary hospital in Rome, Italy, there was no difference overall in the incidence of 3GCR (and CRE) BSI during the COVID-19 period compared to a pre-COVID cohort [9]. However, the frequency of 3GCR *K. pneumoniae* showed a tendency to a higher frequency among pre-COVID compared to COVID-negative patients.

A study in a reference center in Jakarta showed similar findings: the frequency of 3GCR *K. pneumoniae* and *E. coli* did not increase between 2019 and 2020 [40].

On the contrary, a study from four centers in Los Angeles showed a 20% drop in the rate of various infections (BSIs included, number unspecified) due to beta-lactamase-producing bacteria between the first and second quarter of 2020. The Authors impute such a decrease to isolation precautions and a measured increase in alcoholic solution and hand soap consumption during the study period [41]. A retrospective study, conducted between 2017 and 2020, also showed a significant reduction of infections by MDR bacteria (number of BSIs unspecified) for the period March–June 2020, ESBL-producing *K. pneumoniae* being the second most represented pathogen [20]. It was noted that patients with COVID-19 had a higher incidence of infections by MDR organisms compared with non-COVID patients during the pandemic period. This was attributed to several factors: empirical use of antibiotics (even though the immediate effect on a single patient may be disputable), a greater request for microbiological tests by Infectious Diseases physicians compared to other specialists, and the comorbidities of patients hospitalized with COVID-19.

#### 3.3.2. Carbapenem-Resistant Enterobacterales (CRE)

In a wide multicenter study on HAI among patients hospitalized in the ICU in 2020 compared to 2019, Lepape et al. showed that the overall rate of HAI was higher in the SARS-CoV-2 infected group from 2020 than in both the 2019 group and 2020 SARS-CoV-2 uninfected patients [42]. A significantly higher frequency of ESBL-producing Enterobacterales and CRE-causing infection, with no distinction between BSI and other infections, was observed among SARS-CoV-2 infected compared to SARS-CoV-2 uninfected patients for the year 2020.

Consistently, in a small retrospective study including patients admitted to the ICU, with at least one confirmed MDR-gram negative BSI during 2019–2020, non-COVID-19 patients had a higher incidence of MDR-gram negative BSIs and were more likely to present *K. pneumoniae* BSIs compared to COVID-19 patients [43].

In an Italian multi-center study on hospitalized patients with BSIs, the incidence rates of carbapenem-resistant *K. pneumoniae* increased significantly from 2019 to 2020 [44].

On the other hand, in an above mentioned study comparing the years 2019 with 2020 in a reference center in Jakarta, including 1895 patients with positive blood cultures, the authors found that the frequency of carbapenem-resistant *K. pneumoniae* and *E. coli* did not increase [40]. Similarly, in a recent study on hospital-acquired BSI conducted in a reference university hospital in Rome between 2018–2021, comparing pre-COVID, COVID-negative, and COVID-positive patients, the frequency of CR *K. pneumoniae* did not vary before and after the pandemic [9]. Moreover, in a retrospective study conducted in Beirut, Lebanon, the isolation density of CRE-BSI in 2020 reached its lowest value since 2016, with a decrease of 64% in isolation density of CRE BSI/1000 patient days from 2019 to 2020 [31] and in a monocentric Spanish study, Guisado-Gil et al. showed that the incidence of hospital-acquired MDR bacterial BSI had a stable trend, attributed to AMS program in place [45].

### 3.4. Difficult-to-Treat Resistant Pseudomonas aeruginosa (DTR-PA)

*Pseudomonas aeruginosa* with difficult–to-treat resistance (DTR-PA) is defined as a *P. aeruginosa* strain that exhibits non-susceptibility to all of the following: piperacillin-tazobactam, ceftazidime, cefepime, aztreonam, meropenem, imipenem-cilastatin, ciprofloxacin and levofloxacin [46,47,48,49]. Nowadays, it represents one of the biggest challenges in the field of antibiotic resistance [50,51], standing out as a leading cause of mortality due to healthcare-associated infections. In recent years, difficult–to-treat resistance has become a popular idea in the field, as the definition permits to convey treatment-limiting resistance into a more practical approach than other definitions such as carbapenem-resistant *P. aeruginosa*. Moreover, as documented through a single-center retrospective analysis among 1576 patients with gram-negative BSI [52], the definition of DTR instead of XDR (extensively drug-resistant bacteria) or MDR (multidrug-resistant bacteria, defined as nonsusceptibility to at least 1 antibiotic in at least 3 classes for which *P. aeruginosa* susceptibility is generally expected: penicillins, cephalosporins, fluoroquinolones, aminoglycosides, and carbapenems) seemed to be the most appropriate to describe the cases of *P. aeruginosa* infection with limited therapeutic options, identifying it as a useful tool in these challenging cases.

Even before the COVID-19 pandemic started, some studies have tried to define the burden of DTR-PA BSI. For example, a comprehensive analysis conducted by Ramanhatan et al. on a large cohort of 3256 hospitalized adults between 2012 and 2018 with a confirmed diagnosis of PA-BSI showed a prevalence of 11.3% MDR strains: within this group, 18.6% were resistant to all carbapenems tested [53].

On the other hand, another study by Gerver et al. found that, despite an overall increase of DTR-PA isolates in the 2009–2018 decade, the rate of patients dying after *P. aeruginosa* BSI has been globally declining during the pre-COVID era [54].

The CDC pointed out already in 2018 that DTR-PA accounted for at least 32,600 cases and 2700 deaths/year in the USA, considering all kinds of infections. Furthermore, a recent CDC report analyzing the impact of the SARS-CoV-2 pandemic on the prevalence and mortality of MDR germs showed a sharp increase in resistant infections during hospitalization, including a global rise in MDR *P. aeruginosa* infections up to 32% [7].

A recent systematic review analyzed the trends in *P. aeruginosa* bacteremia during the COVID-19 pandemic and the Authors pointed out the inconsistency of the data about the incidence and prevalence of DTR-PA during this period [55]. For example, a study by Amarsy et al. showed an increase in the rate of BSIs caused by MDR agents—including *P. aeruginosa* ceftazidime-resistant strains—since the beginning of the COVID era [16], while other ones claimed the lack of significant variations in the incidence and prevalence of DTR-PA infections—including BSIs—[56,57] despite a diffuse broad-spectrum antimicrobial use witnessed in some hospitals during SARS-CoV-2 worldwide emergency [58].

### 3.5. Carbapenem-Resistant Acinetobacter baumannii (CR-Ab)

*Acinetobacter baumannii* is a glucose non-fermentative, non-motile, catalase-positive, oxidase-negative, and non-fastidious Gram-negative coccobacillus, known as one of the main causes of HAI, especially BSI, Healthcare Associated- and Ventilator-Associated- Pneumonias (HAPs and VAPs) [59]. Due to its ability to survive on surfaces, *A. baumannii* often contaminates medical equipment and the healthcare environment [60], leading to outbreaks in hospital settings, especially in cases of prolonged hospitalization or needing mechanical ventilation and invasive procedures [59]. A cross-country systematic analysis investigating deaths and DALYs attributable to and associated with AMR among the WHO European region and its countries in 2019, revealed that *A. baumannii* was one of the main pathogens responsible for death linked with AMR [61].

Several studies have investigated CR-Ab infection rates before the COVID-19 pandemic. For example, a multicentric, prospective, observational study conducted between 2017 and 2018 in Italy among 12 large tertiary-care hospitals, observed 281 episodes of BSI due to MDR-AB: 98.6% of *A. baumannii* strains were considered XDR and 1.4% PDR (pan drug-resistant). Overall, the 14-day mortality rate was 61.2%, while the 30-day mortality was 73.6% [62]. Moreover, a surveillance study conducted in China on MDR organisms isolated from blood cultures between 2014 and 2019 found up to 70.3% MDR phenotypes when *A. baumannii* was documented, with a prevalence of CR-Ab rate between 55.7% and 71.2% during the surveillance time [63].

Superinfections by CR-Ab have been one of the main concerns during the COVID-19 pandemic, as critically ill patients pool with prolonged ICU staying, the necessity of permanent devices, such as ventilators, central venous catheters or indwelling catheters, and immunosuppressive therapy (corticosteroids and immunomodulators, as tocilizumab or baricitinib) increased.

According to the European surveillance data, *A. baumannii* infections have been more frequently observed during 2020 than in previous years, with higher percentages of CR-Ab isolation and a west-to-east gradient between European countries [4]. This trend has been confirmed also outside Europe, since the rates of hospital-onset of carbapenem-resistant *A. baumanii* cases increased up to 78% in 2020, after a phase of reduction and plateau between 2012–2017 [7]. 

An investigation conducted on HA-BSI in patients deceased with COVID-19 in Italy, identified *A. baumannii* as one of the most frequently isolated pathogens, mainly in ICU, with a 100% of carbapenem resistance profile [64]. Similar results were confirmed in a retrospective observational study conducted in Greece, showing CR-Ab as the principal cause of Gram-negative BSI [65]. In a large teaching hospital in Rome, Italy, there was a significant increase in CR-Ab BSI incidence during the COVID-19 period compared to a pre-COVID cohort, as CR-Ab isolates were responsible for 45% of *A. baumannii* BSI [9]. Corresponding results have also been observed in two retrospective cohort studies conducted both in India and Brazil, assessing again *A. baumannii* as the leading cause of Gram-negative-BSIs in patients hospitalized for COVID-19 [66,67]. Taysi et al. confirmed *A. baumannii* as the leading cause of secondary lower respiratory tract infections in patients hospitalized in ICU for COVID-19, but not for BSI; however, among BSI due to *A. baumannii,* all of the isolated strains were carbapenem-resistant [68].

A Turkish study compared the nosocomial BSI rate between 2018 and 2020, observed in a COVID-19 hospital versus a COVID-free Oncology Centre: the infection density rates for CR-Ab BSI was higher in COVID-hospital rather than in the Oncology Centre, with a significant increase, especially in the third (14.8, 95% CI 9.5–22) and fourth quarters (11.9, 95% CI 7.9–17.3) of 2020 [34].

Interestingly, many studies revealed the association between an increased incidence of colonization and subsequent infections by CR-Ab, during the COVID-19 pandemic.

A before-and-after, cross-sectional study conducted in Bologna, Italy, found an overall increase of colonization by CR-Ab, comparing January–April 2019 versus January–April 2020, with a rising incidence of CR-Ab BSI and lower respiratory tract infections observed in the same period [69]. An association between colonization and infection by CR-Ab has been illustrated in a multicentric observational retrospective study, published in 2022. Among 176 SARS-CoV-2 patients enrolled, 129 (73%) presented an invasive infection with CR-Ab, (VAP in 60.7% of cases, BSI in 26.6%, with a 6.5% of concurrent VAP and BSI). CR-Ab colonization was reported in 165 patients (93.7%), and 118 patients previously colonized developed invasive infections [70].

**Table 1:** Main characteristics of the studies included in the review (arranged by publication year).

Authors (Year) [Bibliography Reference]	Design of the Study (Country)	Setting	Pathogens	**Comparison Group: Non COVID-19 Era or Non-COVID-19 Department**	**Incidence Differences**
Guisado-Gil et al. (2020) [45]	Single center retrospective observational study (Spain)	Not specified	MRSA, VRE, 3GCR, CRE, CR-Ab, CR-PA	Yes	None
Polemis et al. (2021) [35]	Multicenter retrospective observational study (Greece)	Both ICU and non-ICU	MRSA, VRE, 3GCR, CRE, CR-Ab, CR-PA,	Yes	↑ VRE, CR-Ab
Protonotariou et al. (2021) [65]	Single center retrospective observational study (Greece)	Both ICU and non-ICU	MRSA, VRE, 3GCR, CRE, CR-Ab, CR-PA	Yes	↑ VRE, 3GCR, CRE, CR-Ab
Chamieh et al. (2021) [31]	Single center retrospective observational study (Lebanon)	Both ICU and non-ICU	MRSA, VRE, CRE, CR-Ab, CR-PA	Yes	↑ CR-PA; ↓ CRE, VRE, CR-Ab
Polly et al. (2021) [18]	Single center retrospective observational study (Brazil)	Both ICU and non-ICU	MRSA, VRE, 3GCR, CR-Ab, CR-PA	Yes	↑ MRSA and CR-Ab; ↓ CR-PA and CRE
Hirabayashi et al. (2021) [57]	Multicenter retrospective observational study (Japan)	Not specified	MRSA, 3GCR, CRE, CR-PA	Yes	↓ MRSA, 3GCR, CRE, CR-PA
Cole et al. (2021) [41]	Multicenter retrospective observational study (USA)	Not specified	MRSA, VRE, 3GCR	Yes	↓ MRSA, VRE, 3GCR
Bentivegna et al. (2021) [20]	Single center retrospective observational study (Italy)	Not specified	MRSA, 3GCR, CR-Ab	Yes	↑ 3GCR; ↓ MRSA, CR-Ab
Pasquini et al. (2021) [44]	Multicenter retrospective observational study (Italy)	Both ICU and non-ICU	MRSA, CRE, CR-Ab	Yes	↑ MRSA, CRE, CR-Ab
Amarsy et al. (2021) [16]	Multicenter retrospective observational study (France)	Not specified	MRSA, 3GCR	Yes	↑ MRSA, 3GCR
Pascale et al. (2021) [69]	Multicenter retrospective observational study (Italy)	Both ICU and non-ICU	CRE, CR-Ab	Yes	↑ CR-Ab
Afzal et al. (2021) [27]	Single center retrospective observational study (USA)	non-ICU	MRSA	Yes	↑ MRSA
Palanisamy et al. (2021) [66]	Single center retrospective observational study (India)	ICU	CR-Ab	No	NA
Langford et al. (2022) [30]	Systematic review and metanalysis (Europe, America, Asia, Australia)	Not specified	MRSA, VRE, 3GCR, CRE, CR-Ab, CR-PA	Yes	↑ 3GCR, CR-Ab, CR-PA, CRE
Metan et al. (2022) [34]	Single center retrospective observational study (Turkey)	Both ICU and non-ICU	MRSA, VRE, 3GCR, CRE, CR-Ab, CR-PA	Yes	↑ CR-Ab; ↓ CRE, 3GCR
Sinto et al. (2022) [40]	Single center retrospective observational study (Indonesia)	Not specified	MRSA, VRE, 3GCR, CRE, CR-PA, CR-Ab	Yes	None
Giannitsioti et al. (2022) [19]	Single center retrospective observational study (Greece)	non-ICU	MRSA, VRE, 3GCR, CR-Ab, CR-PA	Yes	↑ VRE
Jeon et al. (2022) [32]	Single center retrospective observational study (South Korea)	Both ICU and non-ICU	MRSA, VRE, CRE, CR-Ab, CR-PA	Yes	↑ MRSA, VRE, CRE ↓ CR-Ab
Shbaklo et al. (2022) [17]	Single center retrospective and prospective observational study (Italy)	Both ICU and non-ICU	MRSA, 3GCR, CRE, CR-Ab, CR-PA	Yes	↑ MRSA, 3GCR, CRE, CR-Ab, CR-PA
Lessa da Costa et al. (2022) [67]	Single center retrospective observational study (Brazil)	Both ICU and non-ICU	MRSA, 3GCR, CRE, CR-Ab, CR-PA	No	NA
Monaco et al. (2022) [64]	Multicenter retrospective observational study (Italy)	Both ICU and non-ICU	MRSA, 3GCR, CRE, CR-Ab, CR-PA	No	NA
Lepape et al. (2022) [42]	Multicenter retrospective observational study (France)	ICU	MRSA, 3GCR, CRE	Yes	↑ MRSA, 3GCR, CRE
Cogliati Dezza et al. (2022) [43]	Single center retrospective observational study (Italy)	ICU	CRE, CR-Ab	Yes	↑ Cr-Ab; ↓ CRE,
Fukushige et al. (2022) [36]	Single center retrospective observational study (Taiwan)	Not specified	VRE	Yes	↑ VRE
Ng et al. (2022) [55]	Systematic review (Europe and Asia)	Not specified	CR-PA	Yes	None
Montrucchio et al. (2022) [70]	Multicenter retrospective observational study (Italy)	ICU	CR-Ab	No	NA
Abubakar et al. (2023) [10]	Systematic review (Europe, Asia, America)	Both ICU and non-ICU	MRSA, VRE, 3GCR, CRE, CR-Ab, CR-PA	Yes	↑ MRSA, VRE, CR-Ab, CRE; ↓ 3GCR, CR-PA
Segala et al. (2023) [9]	Single center retrospective observational study (Italy)	Both ICU and non-ICU	MRSA, VRE, 3GCR, CRE, CR-Ab, CR-PA	Yes	↑ MRSA, CR-Ab; ↓ 3GCR
Taysi et al. (2023) [68]	Single center retrospective observational study (Turkey)	ICU	MRSA, 3GCR, CRE, CR-Ab	No	NA

Abbreviations: ICU (Intensive Care Unit), methicillin-resistant *Staphylococcus aureus* (MRSA), vancomycin-resistant *Enterococcus* spp. (VRE), third-generation cephalosporins-resistant (3GCR) and carbapenem-resistant Enterobacterales (CRE), carbapenem-resistant *Acinetobacter baumannii* (CR-Ab), and difficult-to-treat resistant *Pseudomonas aeruginosa* (DTR-PA), not applicable (NA). Up arrow: increased incidence. Down arrow: decreased incidence.

**Table 2:** Challenges to Infection Prevention and Control (IPC) and Antimicrobial Stewardship (AMS).

Potential Healthcare Disruptors	Examples	**Outcome**
Decrease of resources	Understaffing in healthcare pre-existent COVID-19 pandemic. Specialized healthcare workforce diverted to another context (i.e. from gynecology to COVID wards). Microbiological resources focused on virology. No therapy guidance for this new disease/difficult differential diagnosis with bacterial pneumonia in the early phase of the pandemic. No new investments to counter the pandemic overload.	↑ use of inappropriate antibiotic regimens. ↓ AMR surveillance data/specimens.
Increase in critical illness	Delayed access to ICU wards for critically ill patients due to overcrowding. Prolonged hospitalization due to quarantine protocols and post-acute care in the early phase. Increased number of immunosuppressed people (COVID-19-related, or therapy-related).	↑ use of broad-spectrum antibiotics. ↓ AMS protocols compliance.
Decrease in infection control	Emergency settings (camp hospitals, inadequate structures converted in COVID-19 wards). Overcrowded emergency rooms/wards/ICUs. Personal protective equipment shortage/recycling. Lack of proper disinfection, asepsis, and antisepsis. Consequent lack of compliance to IPC measures on MDR pathogens.	↓ IPC protocols compliance. ↑ spread of MDR pathogens.

Abbreviations: ICU (Intensive Care Unit), AMR (antimicrobial resistance), AMS (antimicrobial stewardship), IPC (infection prevention and control), MDR (multidrug resistance). Up arrow: increase. Down arrow: decrease.

## 4. Discussion

In this narrative review, we identified an increasing trend in the prevalence of MDR BSI during the COVID-19 pandemic for VRE, MRSA, and CR-Ab, while data is not unanimous for 3GR Enterobacterales and CRE. As DTR-PA BSI has rarely been reported in the literature, no conclusions can be drawn regarding this pathogen. 

Given that the literature concerning specifically these topics collectively is scarce, this review provides an important initial overview of the microbiological trends in resistance to bloodstream infections, which needs further exploration.

As we have seen in the early phase of the pandemic, severe COVID-19 patients often required ICU hospitalization and had higher rates of bacterial coinfections than the general inpatient population [71,72]. This may reflect the likelihood of ICU patients requiring invasive life-support interventions, such as mechanical ventilation or central intravascular access, notorious risk factors driving healthcare-associated infections, and therefore antibiotic therapies.

However, prescribing practices in COVID-19, especially in the early phase, did not reserve antibiotic therapy only for the critically ill or coinfections: a meta-analysis conducted in 2021, examining the published research on this matter, found that almost 75% of non-ICU inpatients with COVID-19 received antibiotics while the estimated bacterial coinfection prevalence was less than 10% overall [73].

The overuse of antibiotics for treating COVID-19 patients is a sum of several factors. Some reasons are related to the response of medical and non-medical communities to SARS-CoV-2 infection: early panic about an unknown disease, lack of proper awareness of AMR in the public, potential access and affordability to antibiotics without prescription, and use of leftover antibiotics from earlier prescriptions [30,73,74].

Some factors, on the other hand, are closely related to the disease: for example, a similar presentation to pneumonia may have shaped antibiotic consumption, given the mortality risk of bacterial pneumonia [75,76,77]. As the differential diagnosis of bacterial versus viral pneumonia in critically ill and hospitalized patients is challenging [78], clinical assessment and laboratory tests are essential to help discern the necessity of antibiotics and withhold unnecessary prescriptions [79]. In the early pandemic, the bacterial co-infection risk in COVID-19 patients was deeply overestimated, therefore antibiotic therapy became the gold standard in critically ill patients to prevent possible superinfections, derived from the experience of post-influenza bacterial pneumonia.

Notably, another key driver of overuse may be found in the early pandemic and other converging habits. In the beginning, several drugs were used because they were considered presumably effective and even recommended in preventing and treating SARS-CoV-2 infection, including antimicrobial agents such as ivermectin and azithromycin. Based on in vitro studies, showing the antiviral activity of ivermectin against a wide range of RNA and DNA viruses [80], this antiparasitic agent was largely used in trials and clinical practice during the first period of the COVID-19 pandemic [81]. Evidence on whether ivermectin reduces mortality, the need for mechanical ventilation, the need for hospital admission, and time to clinical improvement in COVID-19 patients is of very low certainty and quality, due to the small size and methodological limitations of the available trial data, including a small number of events [82].

While scientific bodies have suggested that azithromycin’s antibacterial and anti-inflammatory properties may be useful in the empirical treatment of pulmonary co-infections in COVID-19 patients, the evidence for its direct activity in COVID-19 has been largely inconclusive [83,84]. Although clinical trials, such as RECOVERY, PRINCIPLE, and COALITION II, showed the lack of efficacy of azithromycin on clinical outcomes [85,86,87], several studies confirmed that azithromycin was the most commonly prescribed antibiotic during the COVID-19 pandemic [88,89,90].

Another major challenge in our search was the high variability in prevalence across countries, along with the heterogeneity of reporting. In addition, the studies often analyzed the AMR phenomenon in BSI during the COVID-19 era as a whole, without a detailed description of the impact of each pathogen.

Overall, the data from the literature are sometimes inconclusive and contradictory.

For example, our overview shows conflicting evidence regarding the impact of MRSA during the pandemic. Trends toward a decreased prevalence in Europe, starting before the pandemic, seemed to continue even during COVID-19, in both noninvasive and invasive samples [61]. However, many reports have pointed out the increased incidence of MRSA BSI in COVID-19 patients [10,19]. The key to understanding this inconsistency may be possibly found by looking at other viral illnesses. It is worth mentioning that superinfections during viral pandemics are a common complication and *S. aureus* is one of the leading pathogens, as in the case of the Spanish flu in 1918–1919 or the H1N1 influenza pandemic in 2009–2010 [91,92]. However, some distinctions must be made as the patterns of infection in influenza differ from those of COVID-19. In fact, during this pandemic, coinfections at baseline were rare while the nosocomial origin stood out as the main source of infection in this pool of patients, which explains the rates of MRSA invasive infections.

The evidence seems more consistent with other bacteria, such as VRE. An explanation for the unexpectedly high frequency of BSIs due to *Enterococcus* species may be the disruption of the gut barrier caused by SARS-CoV-2 [26]. It has also been shown that *Enterococcus* spp. frequently colonize the respiratory tract of intubated patients and are frequently transmitted from one to another, thus increasing the risk of nosocomial infections [74]. An alternative additional explanation is the increased risk of cross-transmission of *Enterococcus* spp. due to possibly relaxed infection-control measures. For example, during the peak of the COVID-19 pandemic, the protection of healthcare personnel from the virus was prioritized even in the context of PPE scarcity, with a possible consequent risk of cross-transmission of bacteria across patients [25]. PPE shortages were so dire that in the early pandemic, especially in the most affected areas, PPEs were worn at the beginning of the work shift with seldom changes throughout, contributing to MDR bacteria spread in the wards. Moreover, the understaffed healthcare workforce was replenished with transferred or undertrained staff, which may have been not entirely familiar with the usual infection prevention procedures and with correct donning/doffing techniques.

According to our findings, increased rates of 3GCR and CR Enterobacterales causing BSI have not been uniformly reported in the literature. Overall, it is arguable that, despite some factors favoring MDR colonization/infection [93,94,95] that are also associated with hospitalization with COVID-19, (such as the need for the ICU, widespread use of broad-spectrum antibiotics, the need for prolonged manipulation of critical patients, the organization of open spaces to facilitate surveillance, the tendency not to replace PPE, and a high turnover of the staff [96,97,98]), other factors may be at play which balance the risk, such as reduced surgical activity and the beneficial impact of correct PPE utilization [39,99,100]. For example, a typical byproduct of the COVID-19 emergency worldwide has been the drop in elective surgery, a notable drive of HAI caused by Enterobacterales, along with non-critical visits, to prevent ongoing SARS-CoV-2 transmission. More studies focusing on infection type and care setting are warranted to explore this topic.

Since a higher rate of BSIs due to MDR Enterobacterales has not emerged from the literature, prompt utilization of broad-spectrum antibiotic therapy seems to be unjustified among patients hospitalized with COVID-19 without adjunctive, specific risk factors and clinical suspicion of a bacterial co-infection.

Data on DTR-PA incidence during the SARS-CoV-2 pandemic turned out to be missing clear evidence, as very few reports and studies have addressed this topic. Thus, further studies are needed to fill this knowledge gap and improve the prevention and management of BSI caused by DTR-PA.

On the other hand, the evidence on CR-Ab BSI that we have collected all points in the same direction: an increase in incidence during the COVID-19 pandemic. *A. baumannii* often contaminates medical equipment and the healthcare environment [60] with its keen ability to form a biofilm, and immunocompromised, critically ill patients are at greater risk of invasive *A. baumannii* infection. Many studies have shown the association between an increased incidence of colonization and subsequent CR-Ab infections [101,102,103,104]. Hence, prolonged ICU stays, invasive devices, and immunosuppressive therapy for the management of critically ill COVID-19 patients have played a pivotal role in the changing epidemiology.

Still, our analysis had some limitations. In accordance with the narrative nature of our review, these results are not immune to selection bias. Second, this overview reflects the published data so far; thus, we could not eliminate any publication bias. For example, another major limitation was the underrepresentation of Australia, and Africa, and more generally, of low-income and middle-income countries, as published evidence in these settings was rare.

The overall quality of the available evidence was not high because our search relied mainly on observational data from case reports, case series, observational studies, and national and international surveillance data.

As AMR is a microbiological trend that needs per se some time to develop, the overuse of antibiotics during COVID-19 may not have immediate consequences on AMR prevalence, but some delay is likely. In addition, AMR investigations rely heavily on national and international surveillance data, which are usually published with a delay of a few years. Changes in antimicrobial epidemiology may not be fully represented by existing AMR surveillance data, as COVID-19 changes in habits may take years to show its consequences on microbiological patterns. The COVID-19 pandemic was characterized on one hand by slowing detection and reporting in surveillance programs, with fewer samples received and tested, and on the other hand, an increase in clinical samples, especially in high-income countries [16]. Therefore, it is important to exercise caution when analyzing these data, as there may be potential biases resulting from changes in patients and testing denominators that are not similar to usual.

In conclusion, our results highlight the necessity of enhancing microbiological surveillance to accurately assess the actual prevalence of AMR in BSI. Strained healthcare systems have faced many obstacles in their routine AMR surveillance during the pandemic, but coordinated action is needed to restore existing programs worldwide. To prevent and reduce the threat of AMR, it is critical to strengthen and invest in tracking and surveillance of AMR. As the COVID-19 pandemic has exposed the fallacies in our healthcare structure and hindered progress made in fighting AMR, antibiotic stewardship efforts must be prioritized, along with investment and research for the development of new antimicrobials to tackle the next pandemic facing humanity, anti-microbial resistance.

## Data Availability

Data sharing is not applicable.
